# Monitoring RNA dynamics in native transcriptional complexes

**DOI:** 10.1073/pnas.2106564118

**Published:** 2021-11-05

**Authors:** Adrien Chauvier, Patrick St-Pierre, Jean-François Nadon, Elsa D. M. Hien, Cibrán Pérez-González, Sébastien H. Eschbach, Anne-Marie Lamontagne, J. Carlos Penedo, Daniel A. Lafontaine

**Affiliations:** ^a^Department of Biology, Faculty of Science, RNA Group, Université de Sherbrooke, Sherbrooke, QC J1K 2R1, Canada;; ^b^Centre of Biophotonics, Laboratory for Biophysics and Biomolecular Dynamics, Scottish Universities Physics Alliance (SUPA) School of Physics and Astronomy, University of St. Andrews, St. Andrews KY16 9SS, United Kingdom;; ^c^Centre of Biophotonics, Laboratory for Biophysics and Biomolecular Dynamics, Biomedical Sciences Research Complex, School of Biology, University of St. Andrews, St. Andrews KY16 9ST, United Kingdom

**Keywords:** single-molecule FRET, transcription, RNA, riboswitch

## Abstract

Cotranscriptional RNA folding is crucial for the timely control of biological processes, but because of its transient nature, its study has remained challenging. While single-molecule Förster resonance energy transfer (smFRET) is unique to investigate transient RNA structures, its application to cotranscriptional studies has been limited to nonnative systems lacking RNA polymerase (RNAP)–dependent features, which are crucial for gene regulation. Here, we present an approach that enables site-specific labeling and smFRET studies of kilobase-length transcripts within native bacterial complexes. By monitoring *Escherichia coli* nascent riboswitches, we reveal an inverse relationship between elongation speed and metabolite-sensing efficiency and show that pause sites upstream of the translation start codon delimit a sequence hotspot for metabolite sensing during transcription. Furthermore, we demonstrate a crucial role of the bacterial RNAP actively delaying the formation, within the hotspot sequence, of competing structures precluding metabolite binding. Our approach allows the investigation of cotranscriptional regulatory mechanisms in bacterial and eukaryotic elongation complexes.

## Significance

Transcription of DNA into RNA is crucial to life, and understanding RNA polymerase (RNAP) function has received considerable attention. In contrast, how the nascent RNA folds into structures that impact transcription itself and regulate gene expression remains poorly understood. Here, we combine single-molecule Förster resonance energy transfer and site-specific fluorescent labelling of transcripts within native complexes to enable real-time cotranscriptional folding studies of a metabolite-sensing riboswitch from *Escherichia coli*. By monitoring the folding of riboswitches stalled at RNAP pausing sites and during active elongation, we reveal a crucial role for RNAP, which directs RNA folding to allow thiamin pyrophosphate sensing within a precise, transcriptional hotspot. Our approach offers a unique opportunity to unveil cotranscriptional processes in eukaryotic and bacterial systems.

The lifecycle of cells inherently relies on the coordination of various processes taking place at the cotranscriptional level (1–4). In most cases, transcription elongation complexes (ECs) act as a nexus for both RNA synthesis and RNA processing, in which external factors intervene at different times and positions of the regulated gene to ensure appropriate biological expression (1). At the heart of cotranscriptional regulation are nascent RNA structures, which have been found to actively control gene expression either by recruiting key cofactors or by modulating the EC elongation rate (1, 5). Accumulated evidence obtained in bacteria and higher organisms indicate that the directional and stepwise synthesis of RNA may kinetically guide the folding of nascent transcripts into structures that are playing important roles in gene regulation mechanisms (1, 3, 6). The short-lived nature of some nascent structures—which are absent in the full messenger RNA (mRNA)—has made it very difficult to characterize their impact on cotranscriptional regulatory mechanisms (3, 6). Recent examples of regulatory mechanisms involving nascent RNA folding have been observed in bacterial riboswitches in which cotranscriptional metabolite sensing dictates the outcome of genetic regulation (3, 7–9). It is very likely that nascent RNA structures contribute to other regulatory mechanisms, since nascent transcripts were shown to participate in several cotranscriptional regulatory processes, such as RNA polymerase (RNAP) II promoter–proximal (10), alternative splicing (11), and bacterial transcriptional pausing (6). The ability to follow in real time the folding of nascent RNA structures within elongating complexes, from transcription initiation to termination, would provide a way to learn about the impact of RNA folding on gene regulation mechanisms (3).

Single-molecule Förster resonance energy transfer (smFRET) assays have been invaluable to study the dynamics of single RNA molecules by allowing the monitoring of specific structural changes under equilibrium or nonequilibrium conditions (12, 13). However, since bacterial and eukaryotic RNAP active sites do not accommodate fluorescent nucleotides (14, 15), studies attempting to monitor the folding of nascent transcripts have been restricted to using synthetic constructs or nonnative systems (12, 16–18). Although such systems provided important insights about transcriptional mechanisms, they do not use the native RNAP and hence cannot establish the influence of key parameters, such as RNAP pausing, elongation rate, and nascent RNA–protein interactions that are specific to native complexes. Consequently, the cotranscriptional insights extracted from these systems cannot be directly extrapolated to a native environment (3).

Metabolite-sensing riboswitches are RNA regulators that directly coordinate ligand binding and regulation by undergoing cotranscriptional folding (19). Riboswitches are comprised of two domains: a highly conserved metabolite-binding aptamer and an expression platform that controls gene expression. Metabolite recognition leads to the regulation of mRNA levels, translation initiation, or splice site selection (19). Interestingly, several lines of evidence suggest that a large proportion of riboswitches operate at the transcriptional level. Indeed, while the majority of *Bacillus subtilis* riboswitches modulate Rho-independent transcription terminators (19), translationally regulating riboswitches in *Escherichia coli* have been suggested to modulate Rho-dependent transcription termination (20, 21), making riboswitches prime examples of cotranscriptional regulators. Unfortunately, because of the transient nature of riboswitch cotranscriptional folding, the impact of short-lived and low-populated RNA structures on the genetic decision is particularly difficult to determine and has never been addressed by smFRET imaging.

Here, we describe an approach allowing us to monitor in real time the folding of nascent transcripts within *E. coli* ECs. The incorporation of both Cy3 and Cy5 dyes within nascent RNA is ensured by initiating transcription with a Cy3-labeled trinucleotide and the site-specific introduction of an azido-uridine analog, which is coupled to a Cy5-alkyne through a click reaction, namely strain-promoted azide-alkyne cycloaddition (SPAAC). We applied this method to study the thiamin pyrophosphate (TPP)–sensing *tbpA* riboswitch within *E. coli* ECs. By imaging ECs stalled at transcriptional pause sites, we observe that nascent transcripts exhibit large variations in TPP binding, which define a sensing hotspot positioned upstream of the translation start codon. We additionally uncover a crucial pause site–dependent role of the RNAP biasing the riboswitch structure toward sensing-competent RNA conformations. Real-time monitoring of actively elongating RNAP revealed an inverse relationship between transcription speed and metabolite-dependent RNA folding, thus indicating that this riboswitch is kinetically controlling translation initiation. We anticipate that this approach will have a broad impact in the study of cotranscriptional RNA folding in other biologically relevant native bacterial and eukaryotic complexes.

## Results

### The Regulatory Mechanism of the *E. coli tbpA* Riboswitch.

**Fig. 1.**
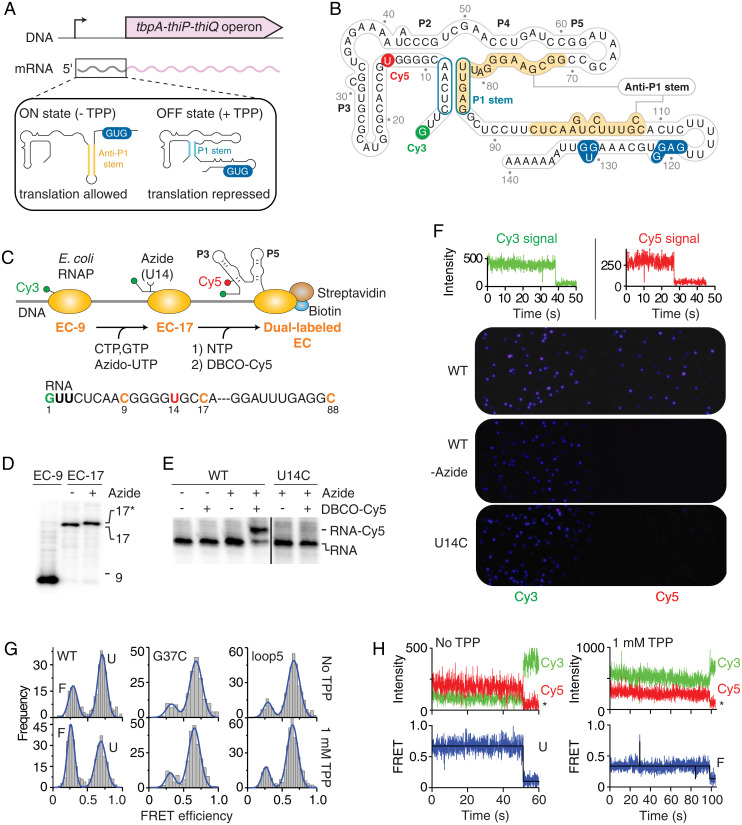
smFRET analysis of *tbpA* transcription ECs. (*A*) Genomic location of the *tbpA* riboswitch. The inset represents the ON and OFF states of the riboswitch and the formation of the anti-P1 and P1 stems, respectively. (*B*) Riboswitch secondary structure in the predicted TPP-bound OFF state. Cy3 and Cy5 dyes are indicated in green and red, respectively. The start codon and the RBS are indicated in blue. (*C*) Schematic approach to obtain Cy3-Cy5 dual-labeled ECs. The sequence of the RNA is shown below. (*D*) Polyacrylamide gel electrophoresis (PAGE) analysis of EC-9 and EC-17 transcripts. A gel retardation is observed for EC-17 with the azide-UTP (17*). (*E*) PAGE analysis of transcripts labeled with DBCO-Cy5 obtained with UTP (−) or azido-UTP (+). A gel retardation is observed for Cy5-labeled RNA species. (*F*, *Top*) Single-molecule traces of directly excited Cy3 and Cy5 show photobleaching in a single step. (*Bottom*) Single-molecule images of fluorescent ECs. While Cy5 fluorescent spots are observed when using wild-type EC (WT), no Cy5 signal is detected without the azido-UTP (-Azide) or using a U14C mutant (U14C). (*G*) smFRET histograms of nascent WT, G37C, and loop5 EC-88 in the absence or presence of 1 mM TPP. The folded (F) and unfolded (U) states are indicated for the WT. (*H*) smFRET time traces of EC-88 in the absence (*Left*) or presence (*Right*) of 1 mM TPP. The anti-correlated Cy3 donor and Cy5 acceptor emission intensities are shown with the resulting FRET trace. Photobleaching events are indicated by an asterisk.

The *tbpA* riboswitch is located upstream of the *thiBPQ* operon encoding an ABC transporter system involved in thiamin and TPP transport (22) (Fig. 1*A*). In contrast to *E. coli* TPP-sensing *thiM* and *thiC* riboswitches (23), very little information is currently available regarding the *tbpA* regulation mechanism. Sequence alignments predicts that the secondary structure of the *tbpA* riboswitch (Fig. 1*B*) is similar to the *thiM* variant (22, 24). Furthermore, the same analysis suggested that TPP binding to the riboswitch results in the sequestration of the ribosome binding site (RBS) and start codon, thereby inhibiting translation initiation (Fig. 1*A*) (22). In the absence of TPP, the formation of the anti-P1 stem ensures ribosome-free access to the RBS, thus allowing the initiation of translation (Fig. 1*A*). Recently, the *tbpA* regulator was also predicted to be involved in small RNA regulation (25) and in antibiotic tolerance mechanisms of *Salmonella typhimurium* (26), suggesting that the riboswitch might operate at multiple regulatory levels.

Although no high-resolution structural detail is available regarding the *tbpA* riboswitch, several biophysical studies of the structurally similar *thiM* variant have revealed that the aptamer is organized around a three-way junction composed of two parallel helical domains (P2 to P3 and P4 to P5) connected to the P1 helix (Fig. 1*B*) (27–29). Crystal structures show that the bound TPP in the *thiM* aptamer is positioned perpendicular to the two helical domains, where distinct RNA pockets contact the extremities of the metabolite (27–29). TPP recognition is ensured through the formation of an intramolecular riboswitch tertiary interaction involving L5 and P3 RNA elements, which are determinant for ligand sensing (30). The recent smFRET analysis of a chemically synthesized *thiM* aptamer showed that the P3–L5 tertiary interaction is not efficiently formed in absence of TPP (31), consistent with existing data (30, 32), suggesting that the *thiM* aptamer domain is largely reorganized upon TPP binding.

### Site-Specific Dual Labeling of Nascent *E. coli* RNAs.

The crystal structure of the *E. coli* RNAP suggests that fluorescent, cyanine-modified nucleotides cannot be accommodated in the RNAP active site (14), implying that they are not efficient substrates for transcription. To circumvent this limitation, we sought to develop an approach allowing the introduction of smFRET dyes within nascent transcripts. In this approach, we use an azido-uridine analog that is readily incorporated into cellular transcripts by endogenous RNAP (33). The site-specific introduction of the azido-UTP analog is achieved using stepwise transcription, the latter allowing the stalling of ECs at any desired position along the DNA by omitting specific nucleotides in the transcription reaction. The azide-modified transcript can then be labeled using copper-free cycloaddition reaction with fluorescent alkyne dibenzo cyclooctyne (DBCO)–Cy5 in native buffer conditions (34).

The first step of our approach consists in initiating transcription with a fluorescent trinucleotide (Cy3-GUU; *SI Appendix*, Fig. S1*A*) and ATP and CTP and UTP to obtain Cy3-labeled ECs stalled at position 9 (Fig. 1*C*, EC-9). Following a round of washing to remove unincorporated nucleotides, the second step is achieved by transcribing with CTP, GTP, and azido-UTP to obtain EC-17 (Fig. 1*C*). The presence of a single uracil that is transcribed from EC-9 to EC-17 ensures the specific incorporation of an azido-UTP analog at U14 (Fig. 1*C*). The third step involves washing and transcribing complexes to a biotin–streptavidin roadblock (7, 35), thus allowing us to obtain cotranscriptionally folded transcripts in the context of stalled ECs. The presence of the biotin at the 5' end of the antisense DNA strand allows for ECs to remain stable on the DNA template (7, 35). Complexes are then coupled to DBCO-Cy5 at U14 using a copper-free click reaction performed under native conditions, yielding Cy3-Cy5–labeled ECs (Fig. 1*C*, dual-labeled EC).

Stepwise transcription reactions were performed using DNA templates containing the lacUV5 promoter fused to the *tbpA* aptamer domain (88 nt). In these control assays, no transcriptional roadblock was used to allow the production of 88-nt nascent transcripts. Specific products were observed for stepwise transcription reactions yielding EC-9 and EC-17 (Fig. 1*D*). When initiating transcription using a fluorescent Cy3-GUU trinucleotide, transcription was found to be unperturbed compared to an unlabeled GUU trinucleotide (*SI Appendix*, Fig. S2*A*). The incorporation of Cy3 in nascent RNAs is consistent with the gel retardation of the 9 nt product when initiating with Cy3-GUU (*SI Appendix*, Fig. S2*A*). Furthermore, the incorporation of the azido-UTP analog was found to be as efficient as UTP in the formation of EC-17 (Fig. 1*D*). Subsequent Cy5 labeling (see *SI Appendix*, Fig. S1*B* for SPAAC reaction) was observed only in the presence of azide-modified transcripts (Fig. 1*E*, WT lanes). Importantly, no Cy5 labeling was observed when attempting a DBCO-Cy5 click reaction using a U14C mutant (Fig. 1*E*), showing that Cy5 labeling is specific to azido-modified residue U14 in the wild-type transcript. These results indicate that Cy3-Cy5 dual-labeled *tbpA* nascent RNAs can readily be obtained.

While developing this technique, we also attempted to directly incorporate a fluorescent nucleotide within *tbpA* nascent transcripts using *E. coli* RNAP. However, transcription reactions performed in the presence of Cy3-CTP did not allow the detection of EC-17 nascent transcripts (*SI Appendix*, Fig. S2*B*). These results emphasize the need to use an azido-UTP analog to introduce Cy5 within nascent RNAs.

### Metabolite Sensing Is Performed by Dual-Labeled Nascent Transcripts.

In principle, the presence of Cy3 and Cy5 dyes could perturb the riboswitch TPP-sensing activity because of steric hindrance. To study riboswitch folding efficiency, we used an RNase H-based assay that monitors ligand-dependent conformational changes (7, 36). This assay relies on the use of a DNA oligonucleotide complementary to a specific riboswitch region whose structure is expected to be modulated upon ligand binding. We designed an oligonucleotide probe targeting the 40- to 49-nt region of the aptamer that is involved in TPP sensing. Nascent 88-nt aptamer transcripts were subjected to RNase H cleavage assays as a function of TPP concentration, and a progressive decrease in RNase H cleavage was observed as the TPP concentration increased (*SI Appendix*, Fig. S3*A*). Fitting the data to a two-state binding model revealed a TPP concentration corresponding to the midpoint of the conformational transition (*K*_switch_) of 227 ± 13 nM (*SI Appendix*, Table S1), suggesting that the *tbpA* riboswitch is well adapted to sense the low-micromolar TPP concentration reported in vivo (37). Importantly, RNase H assays performed on Cy3- or Cy5-labeled nascent transcripts yielded *K*_switch_ values of 218 ± 25 nM and 521 ± 72 nM, respectively, showing less than a 2.7-fold effect on TPP sensing from either Cy3 or Cy5 dye (*SI Appendix*, Fig. S3 *B* and *C*). Thus, these data suggest that both dyes still allow the riboswitch to perform TPP sensing.

### smFRET Imaging of *E. coli* Transcription ECs.

To analyze the conformation of nascent *tbpA* transcripts in the context of *E. coli* RNAP, Cy3-Cy5–labeled 88-nt nascent transcripts within ECs were obtained using the biotin–streptavidin transcription roadblock (Fig. 1*C*). The roadblock was used to anchor ECs on a biotin-functionalized polyethylene glycol surface. Single-molecule imaging was performed using wide-field total internal reflection fluorescence microscopy (TIRFM) (38). Control experiments using Cy3-labeled ECs showed spatially separated emission spots (*SI Appendix*, Fig. S2*C*), where the efficient immobilization of fluorescently labeled ECs was only observed when using intact complexes. For example, the density of fluorescent molecules decreased from ∼190 per field of view (70 × 70 µm) to background levels (≤6) when any of the immobilization reagents (biotin or streptavidin) or labeled EC constituents (Cy3, DNA template, or RNAP) were omitted (*SI Appendix*, Table S2).

Direct excitation of Cy3 and Cy5 dyes of dual-labeled EC showed single-photobleaching steps for each fluorophore, indicating the incorporation of a single Cy3 or Cy5 dye per EC, respectively (Fig. 1*F*, *Top*). In addition, smFRET imaging of ECs (Fig. 1*F*, WT panel and *SI Appendix*, Fig. S2*D*) revealed that the Cy5 fluorescence was not detected in the absence of an azido group (Fig. 1*F*, WT-azide), consistent with Cy5 labeling being specific to the azido group. Furthermore, the use of a U14C mutant revealed no Cy5 signal (Fig. 1*F*, U14C and *SI Appendix*, Fig. S2*D*), showing that the incorporation of the azido-UTP is exclusively performed at position U14 during transcription elongation.

The smFRET analysis of EC-88 complexes showed that nascent transcripts mostly adopted a high-FRET conformation (E_FRET_ ∼0.6) but also folded into a structure exhibiting a lower FRET value (E_FRET_ ∼0.3) (Fig. 1*G*, WT). In the presence of 1 mM TPP, nascent transcripts folded more efficiently into the low-FRET population (Fig. 1*G*, WT), suggesting that it represents the aptamer folded state (F state). The high-FRET state was thus attributed to the aptamer unfolded state (U state) (*SI Appendix*, *Materials and Methods*). As expected, mutants preventing ligand binding (30, 31) (G37C and loop5 mutants; see *SI Appendix*, Fig. S4 for description) did not exhibit an increase of the F state with the addition of TPP (Fig. 1*G*). Lastly, smFRET trajectories showed that nascent riboswitch structures are relatively stable over time with or without TPP (Fig. 1*H*), consistent with a low degree of structural exchange in our conditions.

**Fig. 2.**
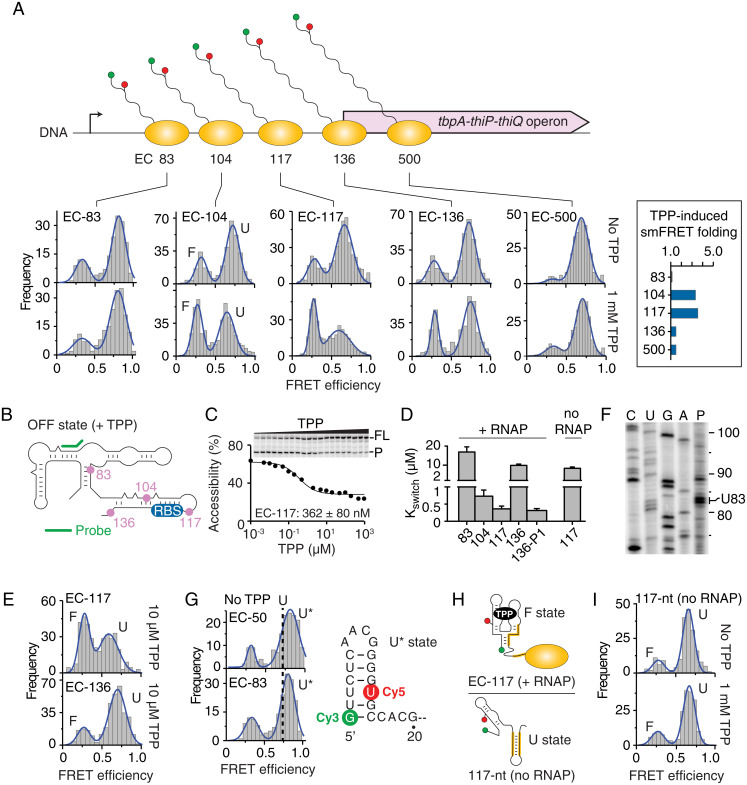
smFRET analysis of *tbpA* nascent transcripts along the transcriptional pathway. (*A*, *Top*) Scheme showing nascent transcripts at transcriptional pause sites (83, 104, 117, and 136) and at position 500. (*Bottom*) smFRET histograms of ECs stalled at specific positions in the absence and presence of 1 mM TPP. The unfolded (U) and folded (F) states are indicated for EC-104. The insert shows the TPP-induced folding ratio for each complex. (*B*) Schematic of the construct used for RNase H probing and the location of the probe (green). Pause site locations within the sequence are shown in pink. (*C*) K_switch_ measurement of EC-117 done in the presence of increasing TPP concentrations. The full-length (FL) and product (P) species are shown on the right of the gel. (*D*) K_switch_ values obtained for various transcripts with (+) and without (−) RNAP. (*E*) smFRET histograms of EC-117 and EC-136 in the presence of 10 µM TPP. (*F*) Mapping of the 83-nt transcriptional intermediate. Reactions were stopped after 45 s (P). (*G*) smFRET histograms of EC-50 and EC-83. The fitted Gaussian shows a population exhibiting a higher-FRET value (U* state) than the U state. A dotted line centered at the maximum of the U state is shown in the histograms. The predicted structure of the U* state is shown. (*H*) Predicted structures of 117-nt nascent transcripts in the presence (*Top*) and absence (*Bottom*) of RNAP. (*I*) smFRET histograms of 117-nt nascent transcripts obtained in the absence and presence of 1 mM TPP.

The structure of nascent *tbpA* transcripts was next monitored within ECs positioned at transcriptional pause sites (7) 104, 117, and 136 (Fig. 2*A*), which are likely biologically relevant, TPP-sensing complexes. Nascent transcripts within EC-104, EC-117, and EC-136 folded into U and F states (Fig. 2*A*, see corresponding histograms), indicating that both states persisted across downstream pause sites. While EC-104 and EC-117 efficiently folded with 1 mM TPP, poor, ligand-induced folding was found for EC-136 (Fig. 2*A*, *Insert*). The close examination of the U state indicates a slight but consistent decrease in the FRET efficiency in the presence of TPP for EC-104 (∼0.65) and EC-117 (∼0.62) when compared to values obtained without TPP (∼0.70 and ∼0.69, respectively). We also observed an increase in the full width at half maximum (FWHM) value of the U state population in the presence of TPP, which is particularly significant when comparing EC-117 without (FWHM ∼0.18) and with TPP (FWHM ∼0.29). None of these changes were observed for EC-88 or EC-136, thus indicating that these variations are specific to EC-104 and EC-117 complexes. These variations of the U state suggest either the presence of an additional TPP-bound structure exhibiting a different E_FRET_ value or another U state involved in partial interactions with TPP. The elucidation of these variations and their potential role in the ligand recognition mechanism will need further investigation.

The TPP binding affinity of each transcription complex was also investigated using RNase H assays, in which transcriptional roadblocks were used to stall RNAP at each pause site (Fig. 2*B*). For example, for EC-117, nascent transcripts were protected from RNase H cleavage at high-TPP concentrations (Fig. 2*C*), which yielded a K_switch_ value of 362 ± 80 nM (*SI Appendix*, Table S1). Moreover, in agreement with smFRET data (Fig. 2*A*), while EC-104 exhibited a similar affinity than EC-117, EC-136 showed a lower TPP-sensing activity (∼30-fold) (Fig. 2*D* and *SI Appendix*, Fig. S5), suggesting that EC-117 represents the last checkpoint for metabolite detection. In-line probing studies also revealed a large difference in binding affinity (∼45-fold) between EC-117 and EC-136 (*SI Appendix*, Fig. S6), which is consistent with our RNase H data. Furthermore, smFRET analysis performed using a physiological TPP concentration (10 µM) (37) showed that nascent transcripts folded with TPP in EC-117 but not in EC-136 (Fig. 2*E*). Accordingly, transcription complexes stalled within *tbpA* open reading frame (ORF) at position 500 showed inefficient TPP-induced folding (Fig. 2*A*, EC-500). We also investigated an unreported transcriptional pause site in the aptamer domain at position 83 (Fig. 2*F*), which did not result in a productive TPP-sensing complex (Fig. 2*D*). A higher-FRET value (E_FRET_ ∼0.8) was measured for EC-83 nascent transcripts, as well as for a shorter construct (EC-50) (Fig. 2*G*), suggesting that these shorter nascent transcripts adopt an alternative hairpin structure (Fig. 2*G*, see U* state). Taken together, our smFRET data suggest that TPP sensing is performed within a narrow hotspot transcriptional window in which *tbpA* nascent transcripts are most responsive to ligand when ECs are located between pause sites 104 and 117.

### *E. coli* RNAP Pausing Impacts Riboswitch Structure and Sensing Activity.

The loss of TPP-sensing activity in EC-136 and EC-500 might result from the formation of the anti-P1 stem (Fig. 1*B*), as observed for the *thiC* riboswitch (7). In agreement with this, mutations disrupting the anti-P1 stem resulted in higher-TPP binding affinity in EC-136 (Fig. 2*D*, 136-P1 mutant). Importantly, although EC-117 contains the sequence required for anti-P1 stem formation (Fig. 1*B*), EC-117 shows very efficient binding properties compared to EC-136 (Fig. 2*E*). These results can be explained by a model in which the RNAP sequesters the 3' sequence of the anti-P1 stem in EC-117 (Fig. 2*H*), thereby allowing F state formation and TPP sensing by hindering the anti-P1 stem. In agreement with our model, the analysis of a 117-nt nascent transcript performed in the absence of RNAP revealed a complete lack of TPP-induced F state (Fig. 2*I*) and a TPP binding affinity reduced by ∼24-fold (Fig. 2*D*, no RNAP and *SI Appendix*, Fig. S7). Supporting evidence of 117-nt reduced ability for ligand sensing was obtained using in-line probing analysis, since only a small variation in structural change was observed upon TPP titration (*SI Appendix*, Fig. S6*A*). The requirement of RNAP for TPP sensing at the pause site 117 clearly indicates the requirement of the transcriptional machinery for metabolite detection.

**Fig. 3.**
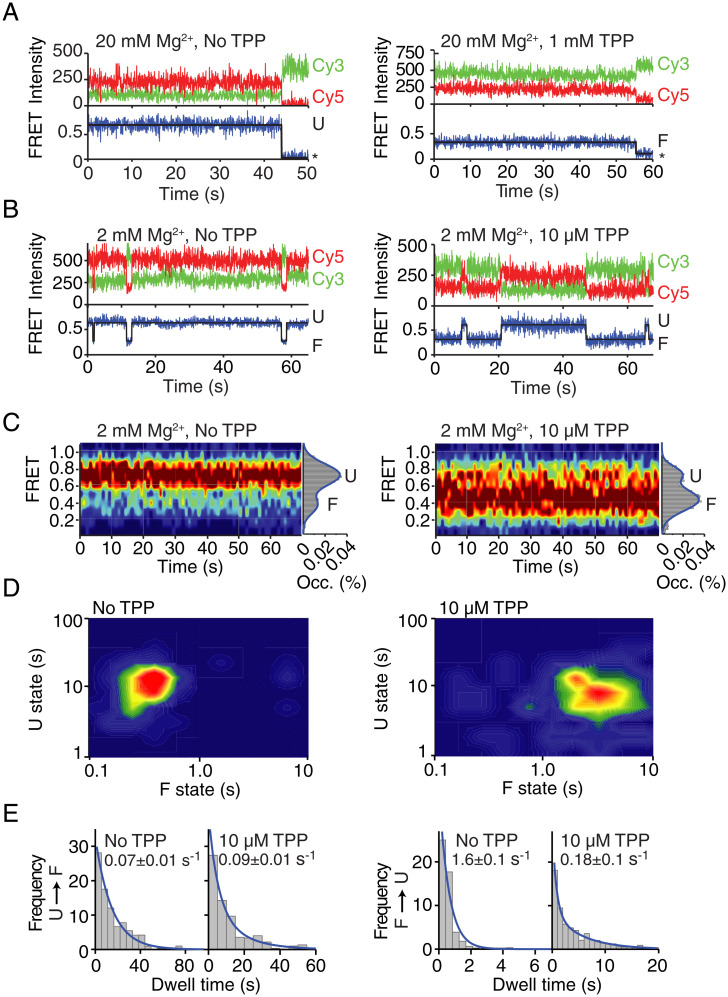
Structural dynamics of nascent transcripts in the context of EC-117. (*A* and *B*) Representative smFRET time traces of EC-117 at 20 mM Mg^2+^ (*A*) in the absence or presence of 1 mM TPP or at 2 mM Mg^2+^ (*B*) in the absence or presence of 10 µM TPP. The anti-correlated donor (Cy3, green) and acceptor (Cy5, red) emission intensities together with the resulting FRET trace are shown. (*C*) smFRET contour plots obtained at 2 mM Mg^2+^ in the absence or presence of 10 µM TPP. Values of 75 single traces were accumulated for the first 70 s. Histograms representing the percentual occupancy (Occ. %) of each state are shown to the right. (*D*) Two-dimensional contour plots of average dwell times for the U and F states at 2 mM Mg^2+^ in the absence or presence of 10 µM TPP. (*E*) Dwell time histograms of the U→F and F→U transitions at 2 mM Mg^2+^ in the absence or presence of 10 µM TPP. Exponential decay values are calculated for each condition.

The TPP-sensing mechanism of *tbpA* nascent transcripts was investigated in the context of the TPP-responsive EC-117. The hidden Markov modeling of traces obtained without TPP showed rare transitions from the U to the F conformer (Fig. 3*A* and *SI Appendix*, Fig. S8*A*). The addition of 1 mM TPP gave rise to long-lived F states (Fig. 3*A* and *SI Appendix*, Fig. S8*B*) indicative of TPP binding. These results also suggest that both conformers exhibit relatively low dynamics during the monitored timeframe (∼1 min). To allow increased structural flexibility of nascent transcripts, we performed smFRET analysis using a lower concentration of Mg^2+^ (2 mM). In absence of TPP, we observed transitions from the U conformer to very short-lived F states (∼0.5 s) that rapidly reverted to the U state (Fig. 3*B* and *SI Appendix*, Fig. S8*C*), confirming that unbound nascent riboswitches can spontaneously access both structures, although with a very strong kinetic bias toward the unfolded conformation. Importantly, when adding 10 µM TPP, our smFRET analysis revealed longer transitions to the F state (Fig. 3*B* and *SI Appendix*, Fig. S8*D*). We calculated the distribution of U and F states, with and without TPP, by accumulating the FRET trajectories from individual molecules across a time window of ∼70 s. The resulting contour FRET plots showed a significant broadening upon addition of 10 µM TPP, reflecting the dynamic equilibrium observed in individual traces (Fig. 3*C*). The single-molecule population histograms displayed a clear shift from the U state with no TPP toward a predominant F state at 10 µM TPP. Taken together, these observations suggest that conformational changes within nascent riboswitches are modulated by metabolite recognition, in agreement with the ability of EC-117 to efficiently sense TPP (Fig. 2*E*).

Contour plot diagrams of average dwell times in the F and U states revealed a very low level of dynamic heterogeneity both with and without 10 µM TPP (Fig. 3*D*). The kinetic analysis of transitions occurring between U and F states indicated that TPP binding to nascent riboswitches decreased the rate of F to U transitions by one order of magnitude, whereas the U to F folding rate remained unaffected (Fig. 3*E*). These data demonstrate that TPP recognition is performed almost exclusively by EC-117 transcripts prefolded into an F state that becomes significantly stabilized upon TPP binding. Together, these observations support a conformational capture ligand–binding model for the TPP riboswitch operating in native complexes, which contrast with 117-nt transcripts that are nonresponsive to TPP ligand (Fig. 2*I*). Thus, our data confirm that RNAP pausing at EC117 delays the formation of structures competing with the hotspot TPP-sensing window, establishing a cotranscriptional ligand recognition mechanism.

### RNAP Elongation Rate Strongly Modulates Metabolite Sensing.

**Fig. 4.**
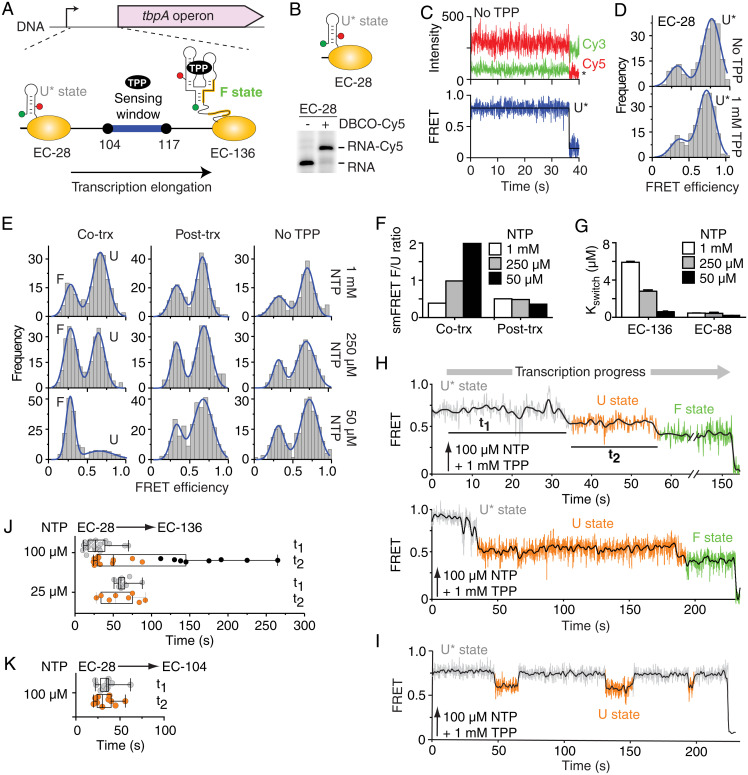
Cotranscriptional TPP sensing by the *tbpA* riboswitch. (*A*) Scheme depicting the 5' untranslated region of *tbpA* with transcriptional pauses. The elongation pathway from EC-28 to EC-136 is shown. The expected locations of Cy3 (green) and Cy5 (red) dyes are shown on the transcript. The TPP sensing window is indicated in blue. (*B*) Predicted structure of the U* state in EC-28. A PAGE analysis of EC-28 transcripts labeled without (−) or with (+) DBCO-Cy5 is shown. (*C*) smFRET time traces of EC-28 in the absence of TPP. The Cy3, Cy5, and FRET time intensities are shown. Photobleaching is indicated by an asterisk. (*D*) smFRET histograms of EC-28 nascent transcripts in the absence or presence of 1 mM TPP. The U* state is indicated. (*E*) smFRET histograms obtained upon transcribing EC-28→EC-136 when TPP (10 µM) was added cotranscriptionally (co-trx), posttranscriptionally (post-trx), or absent. (*F*) smFRET ratios of F to U states for cotranscriptional and posttranscriptional TPP sensing. (*G*) Cotranscriptional TPP binding monitored by RNase H assays for EC-88 and EC-136. (*H* and *I*) Real-time monitoring of the nascent *tbpA* folding. Upward arrows show injection at 5 s. The FRET states and dwell times are shown. (*I*) Fluctuations between U* and U states were observed in some cases. (*J* and *K*) Box plot comparison of t_1_ and t_2_ values obtained from EC-28→EC-136 (*J*) and EC-28→EC-104 (*K*) transcription reactions at indicated NTP concentrations. Black circles represent posttranscriptional events. The extremes, upper and lower distribution quartiles, and the median are represented by whiskers, boxes, and middle lines, respectively.

The presence of a narrow TPP-sensing window between pauses 104 and 117 (Fig. 4*A*) suggests that the *tbpA* riboswitch performs cotranscriptional sensing and that the kinetics of ligand binding and transcription elongation are important for regulation (7, 8). As a result, ligand recognition should be more efficiently performed by slowly elongating ECs, as they spend more time in the sensing window. To directly monitor cotranscriptional TPP sensing, we prepared an EC-28 construct that was readily labeled with DBCO-Cy5 (Fig. 4*B*). smFRET imaging of EC-28 revealed stable FRET signal corresponding to the U* state (Fig. 4*C*), which was not affected by the presence of TPP (Fig. 4*D*). Transcribing the *tbpA* UTR from EC-28 at a slow elongation rate allowed nascent transcripts to sense TPP (Fig. 4*E*, Cotrx; 50 µM NTP). The lower formation of the F state was detected when transcribing at higher-NTP concentrations (Fig. 4 *E* and *F*, see 250 µM and 1 mM), consistent with the elongation rate being crucial for TPP sensing. No change was detected when transcriptions were done without TPP or when TPP was added posttranscriptionally (Fig. 4 *E* and *F*). In agreement with this, a slower rate of transcription elongation was required for efficient TPP sensing, according to RNase H assays (Fig. 4*G*, EC-136). Similar experiments using a shorter template, not allowing the formation of the anti-P1 stem, exhibited very high TPP-sensing activity across NTP concentrations (Fig. 4*G*, EC-88). These results are consistent with the anti-P1 stem being the primary determinant of TPP sensing. Thus, our results strongly suggest that the rate of elongation is central for TPP recognition within native transcription complexes.

### Nascent Transcript Dynamics Measured in Elongating RNAP Using smFRET Imaging.

The folding of nascent riboswitches was next monitored in real-time across the entire EC-28→EC-136 transcriptional window. Upon injecting NTPs and TPP, we found that nascent transcripts followed a specific folding trajectory sequentially transiting from U*, U, and F states (Fig. 4*H* and *SI Appendix*, Fig. S9*A*). Importantly, while the U* to U transition indicates ECs transiting from positions 83 to 88 (Fig. 4*H*), the U to F transition reports metabolite binding to nascent riboswitches. The inspection of traces revealed that the dwell time of the U state exhibits a very large dispersion with values differing more than one order of magnitude between transcripts (Fig. 4*H*, *Upper* and *Lower*). The F state was rarely detected in real-time transcriptions without TPP (<15% of all trajectories) and exhibited a relatively short dwell time (*SI Appendix*, Fig. S9*B*). As a result, we assigned the presence of long-lived F states (∼84 s), observed with TPP to ligand-bound nascent transcripts (*SI Appendix*, Fig. S10*A*). This assignment is consistent with the dwell time values observed for the F state of EC-136, without and with TPP, at steady-state conditions (Fig. 4*E*, 50 µM NTP). Without TPP, 30% of the molecules showed rare interconversions between the U and short-lived F states (*SI Appendix*, Fig. S10*B*) with an average dwell time of ∼6.6 s (*SI Appendix*, Fig. S10*C*). In such conditions, only 6% of EC-136 complexes displayed a long-lived F state. In contrast, the presence of TPP resulted in F states lasting for >100 s without interconversion to U (*SI Appendix*, Fig. S10*D*), thus preventing the estimation of a dwell time value. Together, these data confirm the interpretation of the U to F transition observed in real-time experiments as representing the formation of ligand-bound states.

A subset of real-time transcription traces showed exclusively interconversions between U* and U states (Fig. 4*I* and *SI Appendix*, Fig. S9*C*), suggesting misfolded transcripts unable to perform TPP binding. A similar folding pattern (*SI Appendix*, Fig. S11) was obtained when using an EC-117 riboswitch variant unable to form the P1 stem (P1 stem mutant), consistent with multiple U*–U interconversions being associated with riboswitch misfolding.

The single-molecule kinetic analysis of real-time folding trajectories revealed that the dwell time of the U* state (Fig. 4*H*, t_1_) is dependent on the NTP concentration (Fig. 4*J*), in agreement with RNAP complexes reaching the region 83 to 88 at different elongation rates (*SI Appendix*, *Materials and Methods*). However, we found that the dwell times of the U state (Fig. 4*H*, t_2_) were largely dispersed at 100 µM NTP and exhibited a large fraction (∼40%) of TPP-binding events occurring after >100 s (Fig. 4*J*). We hypothesized that such late binding events are due to RNAP complexes having reached position 136, in which case the presence of the anti-P1 stem prevents the riboswitch to efficiently bind TPP. In agreement with this, a small dispersion of t_2_ values was obtained at a lower rate of elongation (Fig. 4*J*, 25 µM NTP), which favors cotranscriptional sensing at pause sites 104/117 before anti-P1 formation. A similarly small dispersion was obtained at 100 µM NTP when using a shorter template (EC-104) where the anti-P1 stem cannot form (Fig. 4*K*). Together, our real-time analysis of *tbpA* cotranscriptional folding shows that efficient TPP sensing is performed by nascent riboswitches within a narrow transcriptional window defined by the formation of the anti-P1 stem, which is directly related to the rate of transcription elongation. Consequently, our study indicates that the translationally regulating *tbpA* riboswitch relies on the kinetic control of genetic expression, a mechanism that was previously only observed in riboswitches modulating transcription termination (39).

## Discussion

Cotranscriptional RNA folding inherently relies on the sequential and directional polymerization of transcripts emerging from RNAP and may occur on the same timescale of RNA synthesis (40, 41). As a result, the overall speed of transcription can affect transiently adopted structures as well as final transcript conformations (36) and, ultimately, the outcome of associated biological processes. The phage T7 RNAP proceeds five times faster than the *E. coli* RNAP (42) and has been shown in several studies to alter the folding of RNA. For example, it was previously demonstrated that the T7 RNAP-driven in vivo transcription of *rrnB* operon did not lead to the formation of active 70S ribosomes (43), suggesting that the ribosome assembly process requires a fine tuning of the transcription rate of ribosomal RNAs and the assembly process. The use of T7 RNAP was also shown to yield inactive RNAII as a replication primer (44) to introduce new kinetic traps in the *B. subtilis* RNase P RNA (45) and to uncouple metabolite binding from AdoCbl riboswitch folding (36). Cotranscriptional folding was also demonstrated to rely on RNAP pausing, whereby the discontinuous transcription elongation process dictates the structural outcome of emerging nascent RNA (46). Similarly to the speed of transcription, the identity of the polymerase was shown to be important for RNAP pausing (47). For example, while the *E. coli* RNAP efficiently responds to the *his* class-I pause site, the *B. subtilis* RNAP shows a complete inability to pause at the *his* site, suggesting the *E. coli* and *B. subtilis* RNAP profoundly differ in their recognition of regulatory sequences (47). Thus, these results clearly indicate that the study of cotranscriptional RNA folding needs to be performed using the native RNAP (3) [i.e., from the same organism as the investigated RNA molecule (41)].

Several lines of evidence suggest that the approach described here may be applied to study the dynamics of nascent transcripts in the context of other transcription machineries (41). For example, the *Saccharomyces cerevisiae* RNAP II has been shown to initiate promoter-dependent transcription reactions using trinucleotide initiators (48), suggesting that fluorescently labeled trinucleotides could be used to introduce a Cy3 dye at the 5' extremity of the nascent transcript. Furthermore, since *S. cerevisiae* RNAP II transcription ECs support stepwise transcription conditions (48), it indicates that the RNAP II could be positioned to site specifically introduce the required analog. There is a very high probability that an azide-UTP analog would be recognized by the *S. cerevisiae* RNAP II, since it was shown that mammalian RNAPs I, II, and III efficiently incorporate click-compatible UTP analogs to allow subsequent fluorescent labeling (33, 49). This is in agreement with the RNAP core structure being conserved among the three domains of life (50). The latter observation also suggests that the biotin–streptavidin roadblock would be suitable to generate stalled RNAP II ECs, thus enabling the immobilization of such complexes for smFRET analysis. Although there is less available evidence about the feasibility of our approach for human RNAP, current in vitro systems using dinucleotide transcription initiators (51) provide a working system that could be amenable to smFRET analysis. Fluorescent dyes have also been incorporated within RNA molecules through the synthetic reconstitution of bacterial or eukaryotic nucleic scaffolds (52). The fact that both bacterial and eukaryotic RNAP support similar architectures to initiate transcription indicates that the presence of a labeled trinucleotide should be efficiently elongated during transcription.

Our approach allows to site specifically introduce any click-compatible analogs, such as cross-linking reagents or spin labels, for RNA and RNA–protein structural analyses. The stepwise transcription approach described here is highly versatile as it can be used to position the 3' label at any uracil residue located within the RNA 5' region (∼75 nt), which could be further expanded, providing a limited loss of material during transcription steps (53). Alternative preparation methods of ECs (54) will help in expanding the labeling region of nascent transcripts. Nevertheless, the ability to introduce labels within 5' mRNA regions using our technique will allow us to monitor the folding of large RNA structures encompassing the labeled positions, thus enabling us to tackle relatively complex nascent RNA structures (3). The versatility of the approach is also due to RNAP accepting small oligonucleotides to initiate transcription reactions (55), thereby alleviating potential sequence restrictions when preparing initiation complexes using stepwise transcription. Furthermore, in contrast to synthetic nucleic scaffolds (56), our method relies on PCR-made DNA templates, thus providing a fast and inexpensive way to produce dual-labeled nascent transcripts in the kilobase range, a feature that is currently inaccessible using solid-phase chemistry and very challenging when using methods based on enzymatic ligation (57).

Since click-type reactions require simple conditions and a minimum of purification, they constitute an excellent option for the labeling of biological systems (41). Most notorious of all click reactions is the copper-catalyzed alkyne-azide cycloaddition (CuAAC) (58). CuAAC has already extensively been used for the labeling of biological components. However, some problems concerning effects of the reaction on nucleic acids and/or proteins (components of transcriptional complexes) have previously been observed. Notably, copper oxidizes the imidazole found in histidine side chains (59) and reacts with oxygen to generate radicals which can lead to the degradation of nucleic acids (60). However, copper-free SPAAC does not require any catalyst, can be carried out in biological conditions, and therefore represents a more attractive and gentle approach to labeling (61). For these reasons, SPAAC presented a more suitable option for the labeling of nascent RNA within an EC.

The method described here allows us to obtain Cy3-Cy5 dual-labeled bacterial ECs that can be monitored using smFRET at virtually any positions along the transcriptional landscape. Given that our approach is based on the well-established, copper-free click chemistry, it allows us to introduce a biotin group for subsequent purification steps, cross-linking reagents for the characterization of RNA–protein interactions, and spin labels for electron paramagnetic resonance studies. The versatility of our approach suggests that it could be used to study nascent or elongating transcripts in other prokaryotic or eukaryotic transcription machineries (41).

## Materials and Methods

### DNA Oligonucleotides and PCR Designs.

The PCR designs to obtain DNA templates used for transcription assays are listed in *SI Appendix*, Table S4, and the oligonucleotides used are described in *SI Appendix*, Table S5.

### Preparation of Dual-Labeled Transcription ECs.

The *E. coli* RNAP and sigma70 factor were purified as previously described (7). DNA templates for transcription were produced by PCR using oligonucleotides containing the *lacUV5* promoter sequence (*SI Appendix*, Table S4). The formation of stalled ECs was ensured by using DNA templates containing a biotin at the 5' end of the antisense strand. Streptavidin was mixed to a ratio of 5:1 with DNA (7) and added 5 min before transcription initiation. In vitro transcription reactions were performed in three steps to allow the specific incorporation of both Cy3 and Cy5 fluorophores at positions 1 and 14, respectively. Transcription reactions were done in a buffer containing 20 mM Tris·HCl pH 8.0, 20 mM MgCl_2_, 20 mM NaCl, 14 mM 2-mercaptoethanol, and 0.1 mM EDTA. In Step 1 of our procedure, 200 nM DNA template, 600 nM sigma70 factor, and 300 nM RNAP were incubated at 37 °C for 5 min. Transcription reactions were initiated by adding 25 µM Cy3-GUU trinucleotide and 1.25 µM ATP/CTP/UTP nucleotides at 37 °C for 10 min, thus yielding an EC stalled at position 9 (EC-9). The sample was next passed through G50 columns to remove any free nucleotides. In Step 2, reactions were incubated with 2.5 µM CTP/GTP and 2.5 µM azide-modified UTP analog at 37 °C for 5 min to allow the formation of EC-17. Since a single uracil is found at position 14 in the 10- to 17-nt region, it ensures that nascent transcripts carry a single azide at position 14. The Step 3 of the procedure was performed by using G50 columns to wash EC complexes, which were elongated by adding 1 mM NTPs at 37 °C for 1 min, thus yielding stalled complexes at the biotin–streptavidin roadblock. Such stalled complexes were previously shown to be retained during extensive washing procedure (7).

The incorporation of the Cy5 fluorophore at the U14 azide group of nascent transcripts was performed by using copper-free, strain-promoted click chemistry (34). The azide-carrying ECs were incubated with 63 µM DBCO-Cy5 (Jena Bioscience) in the transcription buffer for 1 h at 37 °C. Transcription complexes were then washed twice using G50 columns to remove unreacted DBCO-Cy5 dyes. Dual-labeled ECs were collected in the transcription buffer and were incubated 5 min without or with TPP prior to be used for smFRET analysis.

### Single-Molecule Imaging.

Single-molecule FRET experiments were performed as previously described (62). smFRET traces were recorded from immobilized single ECs using a prism-type total internal reflection setup, including an inverted microscope (Olympus IX71) coupled to a 532-nm laser (Crystalaser) and a back-illuminated Ixon EMCCD camera (Andor). Microscope quartz slides were passivated with a 30:1 mixture of PEG (Laysan Biosciences) and biotinylated PEG. A second passivation round was performed for 30 min using 25 mM MS4-PEG (Thermo Fisher Scientific) diluted in bicarbonate buffer (pH 8.0) to limit protein–surface interactions. A concentration of ∼250 pM dual-labeled ECs was added to the slide. For the imaging of run-off transcripts, 0.2 mg/mL streptavidin was added to the PEG slide to allow immobilization. Data were acquired using a 50-ms integration time on surface-immobilized molecules in the transcription buffer to which was added 2 mM Trolox, 5 mM 3,4-protocatechuic acid (Sigma) and 100 nM protocatechuate dioxygenase (Sigma), pH 7.5, which was used to enhance the photostability of the dye. Donor and acceptor fluorescence emissions were separated using dichroic mirrors (640 DCLP dichroic mirror, Chroma Technology) and imaged onto the left (donor) and right (acceptor) half-chip of the electron multiplying charge-coupled device (EMCCD) camera. The setup allowed recording Cy3 and Cy5 signals simultaneously. Left and right images were corrected for optical aberrations and inhomogeneous evanescence wave illumination using custom-build routines using IDL software (Exelis). Subsequently, an IDL-mapping algorithm was used to correlate the position of Cy3 and Cy5 signals from the same immobilized molecule, and a time-dependent trajectory was built for each dye and for each molecule. The labeling efficiency was quantified at the single-molecule level by calculating the number of spots showing colocalizing Cy3 and Cy5 signals. The labeling efficiency typically ranged between 65 to 70% for analyzed constructs. An example of a single-molecule image is shown in *SI Appendix*, Fig. S2*D*, in which 53 out of 77 spots are found to colocalize in Cy3 and Cy5 channels, thus representing a labeling efficiency of 69%.

### Real-Time smFRET Assays of Cotranscriptional Folding during Transcription Elongation.

Real-time analysis was performed using a setup similar as previously described (62) but included minor modifications to the microscope slide to allow real-time injection during imaging. For instance, 40-mm cover slides were used to allow the fixation at one end of the slide channel of a yellow 200-µL pipette tip, which was cut to be used as a buffer reservoir. In addition, a 18G1 needle (BD) was fixed on the other side of the slide channel. A syringe was attached to a flexible tube connected to the needle to draw off buffer from the reservoir. For each experiment, 100 µL 1:1 solution of transcription and imaging buffer was injected to fill the imaging channel. Injections in real-time assays were carried out at 5 s, unless stated otherwise, after movie data collection was started to have a preinjection FRET level reference. Real-time transcription movies were taken for a sufficiently long time window (>1,000 to 3,000 frames/movie depending on conditions) to ensure that Cy5, Cy3, or both signals were significantly photobleached by the end of the movie. The observation of single-step Cy5 or Cy3 photobleaching, together with no significant changes in the average total emission, was taken as evidence for fluctuations in FRET signal arising from single ECs. Single-molecule FRET trajectories were calculated as indicated before, and only those fulfilling the previous criteria of singleness were considered for data analysis. To help visualization, a smoothing line was added to the experimental FRET trajectory using an adjacent-averaging algorithm with *n* = 5 points in the moving window.

## Data Availability

All study data are included in the article and/or *SI Appendix*.
